# Superoxide increases angiotensin II AT1 receptor function in human kidney‐2 cells

**DOI:** 10.1002/2211-5463.12148

**Published:** 2016-11-16

**Authors:** Mohammad Saleem, Indira Pokkunuri, Mohammad Asghar

**Affiliations:** ^1^ Pharmacological and Pharmaceutical SciencesHeart and Kidney InstituteCollege of PharmacyUniversity of HoustonTXUSA

**Keywords:** AT1 receptor, blood pressure, kidney, oxidative stress, reactive oxygen species, transcription factor

## Abstract

The redox‐sensitive Sp family transcription factor has been linked to the regulation of angiotensin II type 1 receptor (AT1R). However, the exact mechanism of AT1R regulation in renal cells is poorly understood. We tested the specificity of reactive oxygen species (ROS), superoxide vs. hydrogen peroxide (H_2_O_2_), and the specific role of Sp3 transcription factor, if any, in the regulation of AT1R in human kidney cells (HK2 cells). Superoxide dismutase (SOD) inhibitor diethyldithiocarbamate (DETC), but not H_2_O_2_ treatment, increased fluorescence levels of superoxide probe dihydroethidium (DHE). H_2_O_2,_ but not DETC, treatment increased the fluorescence of the H_2_O_2_‐sensitive probe dichloro‐dihydro‐fluorescein (DCFH). These data suggest that SOD inhibition by DETC increases the superoxide but not H_2_O_2_ and exogenously added H_2_O_2_ is not converted to superoxide in renal cells. Furthermore, DETC, but not H_2_O_2_, treatment increased nuclear accumulation of Sp3, which was attenuated with the superoxide dismutase (SOD)‐mimetic tempol. DETC treatment also increased AT1R mRNA and protein levels that were attenuated with tempol, whereas H_2_O_2_ did not have any effects on AT1R mRNA. Moreover, Sp3 overexpression increased, while Sp3 depletion by siRNA decreased, protein levels of AT1R. In addition, Sp3 siRNA in the presence of DETC decreased AT1R protein expression. Furthermore, DETC treatment increased the levels of cell surface AT1R as measured by biotinylation and immunofluorescence studies. Angiotensin II increased PKC activity in vehicle‐treated cells that further increased in DETC‐treated cells, which was attenuated by AT1R blocker candesartan and SOD‐mimetic tempol. Taken together, our results suggest that superoxide, but not H_2_O_2_, via Sp3 up‐regulates AT1R expression and function in the renal cells.

**Abbreviations**
3‐AT: 3‐amino‐1,2,4‐triazole
AT1R: angiotensin II type I receptor
DCFHDA: 2′,7′‐dichlorodihydrofluorescein diacetate
DETC: diethyldithiocarbamate
DHE: dihydroethidium
DMEM/F12: Dulbecco's modified Eagle's medium
H_2_O_2_: hydrogen peroxide
HK2: human kidney 2 cells
PKC: protein kinase C
ROS: reactive oxygen species
SOD: superoxide dismutase

The renal angiotensin II type 1 (AT1) receptor, one of the key components of the renin–angiotensin system, is known to be involved in regulation of blood pressure 1. AT1 receptor, in the presence of angiotensin II, stimulates protein kinase C (PKC) by coupling to Gq protein to initiate critical signaling cascades 2. Higher AT1 receptor function is also reported to occur in hypertension and other cardiovascular disorders 3, 4, 5, 6. Several studies have suggested the involvement of oxidative stress in higher AT1 receptor function 4, 5, 7. However, the mechanism for this is largely unknown.

It is well established that moderate levels of reactive oxygen species (ROS) are critical to the maintenance of cell and organ tissue functions 8, 9. But, a progressive increase of ROS leads to pathophysiological conditions such as hypertension 4, 5, 10, 11, 12, 13. A number of studies have suggested that superoxide and H_2_O_2_ are probably the main ROS that contribute to oxidative stress, which results in hypertension 14, 15, 16, 17. In contrast, some other reports suggest that H_2_O_2_ attenuates hypertension 18, 19, 20. Therefore, clarifying the role of individual ROS in the regulation of renal AT1 receptor function and blood pressure is important.

Sp3 is a redox‐sensitive transcription factor regulating many genes involved in cell signaling, cell growth, and inflammation 21, 22, 23. A number of studies have suggested that elevated ROS modulate the function of Sp3 transcription factor 14, 23. Interestingly, ROS have been shown to up‐regulate AT1 receptor function in hypertension 24, 25. Actually, AT1 receptor promoter possesses the consensus sequence for Sp3 transcription factor 26. However, the mechanistic link between ROS, Sp3, and the up‐regulation of renal AT1 receptor, if any, has not been explored. Therefore, present studies were designed in a human kidney HK2 cell line to test the role of superoxide and H_2_O_2_ in the regulation of Sp3‐mediated AT1 receptor function.

## Materials and methods

### Cell culture

HK2 cells, a proximal tubular cell line from adult human kidney, were obtained from American Tissue Culture Collection (ATCC^®^ CRL‐2190™; ATCC, Manassas, VA, USA). Cell passages between 4 and 17 were used in the study. Three new stock cultures of HK2 from ATCC and thereafter two passages from each stock were used for the experiments (*N* = 6). Cells were cultured as described previously 27. Briefly, cells were cultured in Dulbecco's modified Eagle's medium (DMEM/F12) supplemented with epidermal growth factor (EGF, 10 ng·μL^−1^), bovine pituitary hormone (BPE, 15 μg·mL^−1^), and bovine serum (10% vol/vol) at 37 °C in a humidified incubator with 5% CO_2_. HK2 cells (90–95% confluent) were starved for 1–2 h in DMEM/F12 media without serum, EGF, and BPE, and used for all the experiments unless otherwise stated.

### Superoxide and H_2_O_2_ measurements

Cells (80 000 cells) were suspended in Krebs–Henseleit (KH, pH 7.4) buffer, and incubated with superoxide probe dihydroethidium [DHE; 25 μm (Life Technologies, Eugene, OR, USA)] for 5 min at room temperature. Thereafter, cells were treated with SOD‐inhibitor DETC (500 μm, 5 min) and exogenous H_2_O_2_ (50 μm, 5 min) in the absence and presence of tempol (1 mm). Tempol was added 10 min before adding DETC and remained there in the reaction. DHE fluorescence signal was measured immediately using excitation (490 nm) and emission (610 nm) wavelengths in a spectrofluorometer (Varioskan; Thermo Scientific, Rockford, IL, USA). DHE fluorescence was measured for 30 min at 5‐min time intervals and no significant difference among the readings was found. Data presented were at 5 min.

To measure H_2_O_2_ levels, cells (80 000 cells) were suspended in KH buffer and incubated with 10 μm dichloro‐dihydro‐fluorescein diacetate (DCFHDA, Life Technologies) probe for 30 min at room temperature. Subsequently, cells were treated with H_2_O_2_ (50 μm) and DETC (500 μm) for 30 min and with 3‐amino‐1,2,4‐triazole [(3‐AT) 10 mm)], a catalase inhibitor, for 60 min. DCFHDA fluorescence signal was recorded immediately using excitation (490 nm) and emission (520 nm) wavelengths as mentioned above.

### Measurement of toxicity in DETC‐, tempol‐, and H_2_O_2_‐treated HK2 cells

Toxicity was determined by a colorimetric assay using a commercially available kit (CellTiter 96^®^ Aqueous One Solution Assay; Promega, Madison, WI, USA) and following the manufacturer's instructions. Adherent cells were treated with DETC (500 μm, 2 h), and H_2_O_2_ (50 μm, 30 min) separately, in the absence and presence of tempol (1 mm). Pretreatment of tempol was carried out for 10 min before adding DETC, which remained there with DETC. Cells were suspended in KH buffer and were loaded equally in a 96‐well plate. Subsequently, CellTiter96^®^ AQ_eous_ One Solution Reagent (20 μL) was added to each well, and incubated for 2 h at 37 °C and absorbance was read at 490 nm where the resultant color was directly proportional to the number of viable cells.

### Isolation of nuclear proteins

Adherent cells were treated with DETC (500 μm, 2 h) and H_2_O_2_ (50 μm, 30 min) separately, in the absence and presence of tempol (1 mm). Pretreatment of tempol was carried out for 10 min before addition of DETC and H_2_O_2_. Tempol was present throughout the reaction periods. Control cells were treated with vehicle (water). The reaction was stopped by aspirating media and nuclear proteins were isolated using a kit following the manufacturer's instructions (NE‐PER kit; ThermoFisher Scientific, Rockford, IL, USA). To check the purity of nuclear fractions, GAPDH, a cytoplasmic marker, was determined in cytosolic and nuclear fractions of HK2 cells. Western blotting of GAPDH revealed its absence in the nuclear fraction (Fig. S1).

### Cells transfection with Sp3 plasmid and Sp3 siRNA

A 2.3 kb Sp3 construct cloned into pCMV vector (a generous gift from Dr. Douglas A. Weigent, University of Alabama, Birmingham, AL, USA) was used to overexpress Sp3 in HK2 cells. FuGENE^®^6 (Promega Corp.) was used as the transfection reagent and the manufacturer's instructions were followed. Briefly, cells were grown up to 65% confluency in a six‐well plate and transfections were carried out for 24 h with 1 μg Sp3 vector. For Sp3 siRNA transfection, cells were transfected with 100 ng of Sp3 siRNA for 24 h. Control cells were separately transfected either with empty pCMV vector for Sp3 overexpression experiments or AllStars negative control siRNA (Qiagen, Valencia, CA, USA) 26 for Sp3 siRNA transfection. Thereafter, cells were washed with PBS and lysed with lysis buffer (20 mm Tris/HCl pH 7.5, 150 mm NaCl, 1 mm EGTA, 2.5 mm sodium pyrophosphate, 1 mm glycerol 2‐phosphate, 1 mm Na_3_VO_4_, 1 mm PMSF, and protease inhibitor cocktail) for 10 min on ice. Furthermore, cells were sonicated on ice for 3 s. Cell lysates were used to determine Sp3 and AT1 receptor proteins by immunoblotting.

### AT1 receptor antibody validation

To rule out nonspecific binding of AT1R antibody [# sc 1173; Santa Cruz Biotechnology Inc. (SCBT), Dallas, TX, USA] used in these studies, HK2 cells were transfected with 50 nm AT1R siRNA (Thermo Fisher Scientific Cat # 4392420) with Lipofectamine RNAiMAX transfection agent (Life Technologies, Grand Island, NY, USA) following the manufacturer's protocol. After 48 h of transfection, proteins were isolated and analyzed by western blotting. AT1R expression with siRNA treatment was simultaneously verified by mRNA analysis using qRT‐PCR (data not shown).

### Western blotting

Western blotting was performed using our published method 27. Cell homogenate and nuclear samples (15 μg) were resolved on 8–16% Tris‐glycine gels (ThermoFisher Scientific) by SDS/PAGE. Gels were transblotted onto polyvinylidene fluoride membrane for 2 h at 4 °C. Thereafter, membranes were blocked with 5% BSA in TBST at room temperature (RT) for 1 h and probed with respective primary antibodies in TBST overnight at 4 °C. After washing with TBST, membranes were incubated with corresponding secondary antibodies conjugated with horseradish peroxidase [(HRP) (SCBT)]. Chemiluminescent reagent was used (SCBT) to visualize protein bands using G:BOX image station (Syngene, Frederick, MD, USA). Protein bands were quantified using software integrated with the image station. Primary antibody dilutions used were 1 : 500 for Sp3, AT1R (both from SCBT), and histone 3 (nuclear loading control; Abcam, Cambridge, MA, USA), while 1 : 1000 for GAPDH (cell homogenate loading controls; Millipore, Temecula, CA, USA).

### AT1 receptor mRNA and proteins

Cells were treated with different drugs as mentioned in the Isolation of nuclear proteins section. Cellular RNA was purified using Qiagen mini kit (Qiagen). As per the MIQE (Minimum Information for Publication of Quantitative Real‐time PCR experiments) accepted guidelines, a constant amount of RNA (1 μg) from all samples was reverse transcribed to cDNA using Advantage‐RT‐for‐PCR kit (Clontech Laboratories, Mountain View, CA, USA). Obtained cDNA was diluted 5 times and 9 μL of it was used to set up the qPCR reaction. TaqMan human‐specific primers for AT1 receptor (ID#Hs00258938‐m1) and 18S ribosome internal control (ID# Hs99999901‐s1) were purchased from Life Technologies. The assay mixture consisted of 10 μL TaqMan^®^ Gene Expression Master Mix, 1 μL AT1 receptor or 18S TaqMan primer, and 9 μL of diluted cDNA in a final volume of 20 μL. Reactions were performed in Applied Biosystems^®^ 7300 Real‐Time PCR system using the manufacturer's software. AT1 receptor mRNA was quantified by relative quantification using Delta‐Delta Ct method 28. In the second set of vehicle (water)‐ and drug‐treated cells, AT1 receptor protein levels were determined as above.

### Immunofluorescence

HK2 cells were grown on glass chambered slides (ThermoFisher Scientific) and treated with DETC in the absence and presence of tempol as above. Control cells were treated with vehicle (water). Thereafter, cells were washed with cold PBS, fixed with cold formalin for 20 min and blocked with 5% normal goat serum (NGS) for 1 h at RT. Cells were incubated with AT1 receptor primary antibody (SCBT) in 1% NGS overnight at 4 °C. After washing, cells were incubated with Alexa‐488 secondary antibody for 1 h at RT in the dark. Cells were mounted with DAPI mount media and scanned under a microscope (Olympus Life Science, Waltham, MA, USA) using the green filter. The AT1 receptor antibody used recognizes extracellular loop of the receptor.

### Isolation of cell surface proteins

Cell surface proteins were biotinylated using EZ‐LINK Sulfo‐NHS‐SS‐biotin and isolated with streptavidin magnetic beads (ThermoFisher Scientific). Briefly, cells were treated with DETC (500 μm, 2 h) in the absence and presence of tempol (1 mm), washed twice with cold PBS and incubated with EZ‐LINK Sulfo‐NHS‐SS‐biotin (250 μg·mL^−1^) for 30 min at 4 °C while shaking. Thereafter, glycine (100 mm in PBS) was added to stop the reaction. Cells were centrifuged at 500 ***g*** for 4 min. Cells pellet was lysed with 500 μL lysis buffer (50 mm sodium phosphate buffer (pH 8.0), 150 mm NaCl, 1% Nonidet P‐40, 0.5% Triton X‐100, 1 mm PMSF and protease inhibitor cocktail) followed by sonicating on ice. The cell lysate was incubated with streptavidin‐coated magnetic beads (0.5 mg/50 μL) overnight with end‐over‐end (360°) shaking at 4 °C. Biotinylated proteins–streptavidin magnetic bead complex was separated using magnetic stand (DynaMag‐Spin; Invitrogen, Oslo, Norway). Supernatant was carefully removed and magnetic bead complex was washed with lysis buffer. The biotinylated proteins–avidin complex was dissociated with 40 μL Laemmeli buffer (125 mm Tris/HCl pH 6.8, 4% SDS, 20% glycerol, 5% β‐mercaptoethanol) by heating at 90–95 °C for 10 min. After vortexing, magnetic beads were separated as above and were discarded and the supernatant (30 μL) was used to determine AT1R proteins by western blotting as above.

### PKC activity

Cells were pretreated with tempol (1 mm) and AT1 receptor blocker candesartan (1 μm), separately, for 10 min followed by treatment with DETC (500 μm, 2 h). DETC treatment in the absence of tempol and candesartan was also carried out. Control cells were treated with vehicle (DMSO). Thereafter, cells were treated with angiotensin II (1 μm, 10 min). Cells were taken in lysis buffer (mm: 20 MOPS, 50 β‐glycerolphosphate, 50 sodium fluoride, 1 sodium orthovanadate, 5 EGTA, 2 EDTA, 1% Triton‐100X, 1 dithiothreitol, 1 PMSF, and protease inhibitor cocktail) and passed 4–5 times through a 23‐gauge needle. PKC activity in cell homogenate (10 μg) was measured using an ELISA‐based assay kit (Enzo Life Sciences, Farmingdale, NY, USA).

### Protein estimation

Cellular and nuclear proteins were measured using a Bicinchoninic Acid (BCA) assay kit (ThermoFisher Scientific). Protein concentrations in the samples were determined using BSA standards.

### Statistics

Data are presented as means ± SE. One‐way ANOVA followed by Newman–Keuls *post hoc* test was used to compare the differences among the groups. Student's *t*‐test was used wherever appropriate. graphpad software (GraphPad Software ver. 5; San Diego, CA, USA) was used to analyze the data. The minimum level of significance was considered at *P* < 0.05.

## Results

### Toxicity measurement

**Figure 1:**
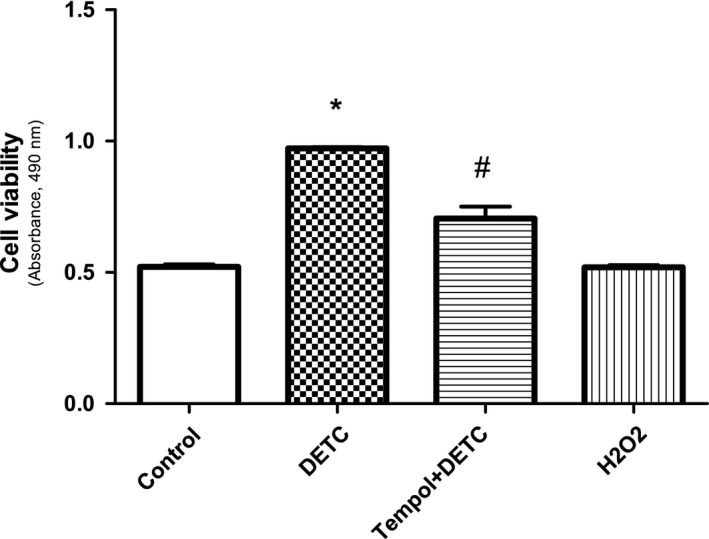
SOD‐inhibitor DETC and H_2_O_2_ do not cause toxicity in HK2 cells: Cells were treated with DETC (500 μm, 2 h), H_2_O_2_ (50 μm, 30 min), and with tempol (1 mm, 2 h). Cells were pretreated with tempol for 10 min before adding DETC. Commercial kit, as described in Methods, was used to detect cell toxicity. Absorbance at 490 nm, directly proportional to the cell viability, was plotted as a bar graph. *N* = 4 separate experiments. *P* < 0.0001, *^#^significantly different from control.

Viable cell number did not decrease and hence toxicity did not increase with DETC, tempol, or H_2_O_2_ treatment in HK2 cells (Fig. 1).

### Effects of SOD‐inhibitor DETC and H_2_O_2_ on the levels of DHE and DCFHDA fluorescence in HK2 cells

**Figure 2:**
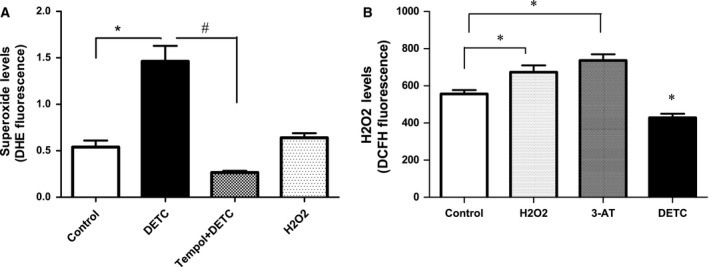
DHE detects superoxide, while DCFHDA detects H_2_O_2_ in HK2 cells: (A) Suspended cells were loaded with DHE probe (25 μm, 5‐min incubation) followed by treatment with SOD‐inhibitor DETC (500 μm, 5 min) in the absence and presence of tempol (1 mm) and H_2_O_2_ (50 μm, 5 min), and DHE fluorescence levels were measured. Bars represent results as mean ± SEM. *N* = 4–6 separate experiments. *P* < 0.0001, *significantly different from control, ^#^significantly different from DETC plus tempol. (B) Cells were loaded with DCFHDA probe followed by treatment with H_2_O_2_, catalase inhibitor 3‐AT, and DETC, and DCFHDA fluorescence levels were measured. Bars represent results as mean ± SEM. *N* = 6 separate experiments. *P* < 0.0001, *significantly different from control (for detailed description about drug treatments, DHE and DCFHDA fluorescence measurements, please see the Methods section).

Diethyldithiocarbamate, but not H_2_O_2_, treatment significantly increased DHE fluorescence levels compared to vehicle‐treated cells (Fig. 2A). In contrast, H_2_O_2_ and catalase inhibitor 3‐AT, but not DETC, significantly increased DCFHDA fluorescence levels compared to vehicle‐treated cells (Fig. 2B). Pretreatment of cells with tempol (SOD‐mimetic) abolished DETC‐mediated increase in DHE fluorescence levels (Fig. 2A).

### Effect of SOD‐inhibitor DETC and H_2_O_2_ on nuclear accumulation of Sp3 protein in HK2 cells

**Figure 3:**
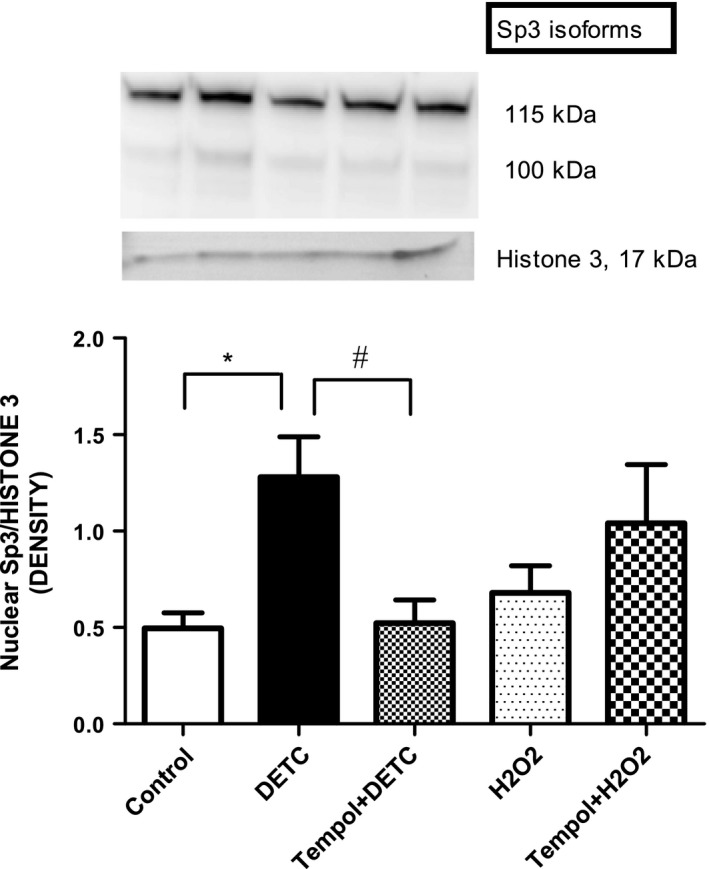
SOD‐inhibitor DETC increases nuclear Sp3 accumulation in HK2 cells: Cells were treated with DETC (500 μm, 2 h) and H_2_O_2_ (50 μm, 30 min) in the absence and presence of tempol (1 mm, 2 h, cells were pretreated with tempol for 10 min), nuclear proteins were isolated, and Sp3 levels were determined by western blotting (details in Methods). Upper panel: representative blot of Sp3 and nuclear protein loading control histone 3. Lower panel: bars represent ratios of the densities of Sp3 and histone 3 proteins bands. Both the Sp3 protein bands were considered for quantification. Results are mean ± SEM. *N* = 5, *P* < 0.0003, *significantly different from control, ^#^significantly different from DETC.

Diethyldithiocarbamate treatment significantly increased nuclear levels of Sp3 protein compared to control cells. This effect was attenuated with tempol treatment. H_2_O_2_ did not show any effect on nuclear accumulation of Sp3 protein (Fig. 3).

### AT1 receptor antibody validation in HK2 cells

**Figure 4:**
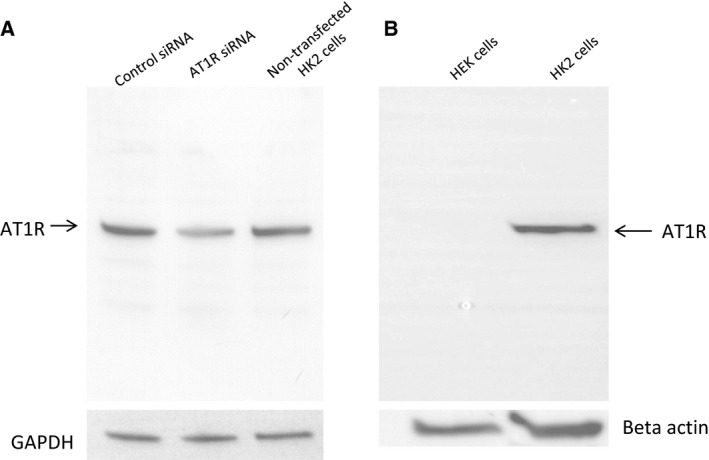
AT1 receptor antibody (SCBT, catalog # SC‐1173) detects AT1 receptor proteins in HK2 but not HEK cells: (A) AT1 receptor expression knockdown in HK2 cells by siRNA: Whole cell lysates were prepared from control siRNA, AT1R siRNA‐transfected, and nontransfected‐HK2 cells, and AT1 receptor expression was analyzed by western blotting as described in Methods. Upper panel: AT1 receptor representative blot. Lower panel: representative blot for loading control GAPDH. (B) AT1 receptors in HEK and HK2 cells: whole cell lysates were prepared from HEK and HK2 cells and AT1 receptor expression analyzed by western blotting as described in Methods. Upper panel: representative blot for AT1 receptor. Lower panel: representative blot for protein loading control beta actin. *N* = 3 separate experiments.

The AT1 receptor antibody (sc‐1173) detected reduced levels of AT1R proteins in the AT1R siRNA‐transfected compared to the control siRNA‐transfected and nontransfected cells (Fig. 4A). AT1R proteins were also not detected by the antibody in HEK cells, which do not express AT1R 29, compared to the HK2 cells (Fig. 4B).

### Effects of SOD‐inhibitor DETC and H_2_O_2_ on AT1 receptor mRNA in HK2 cells

**Figure 5:**
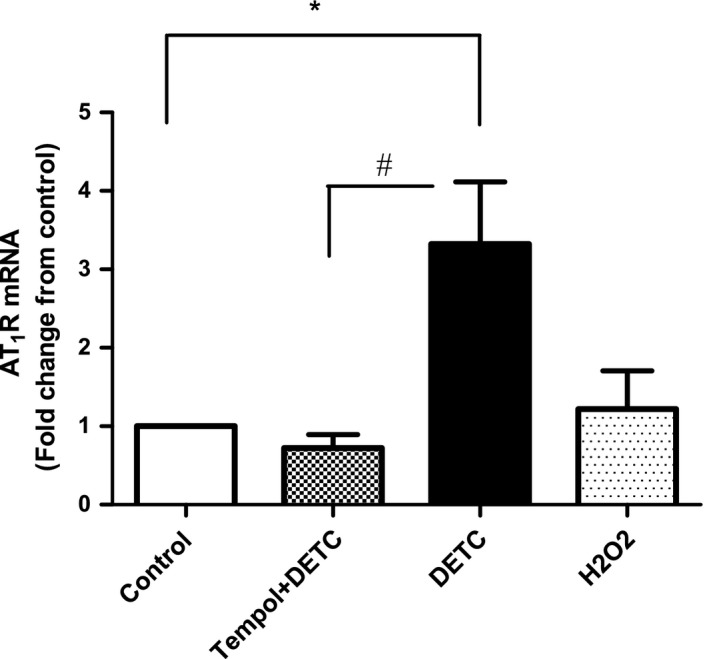
SOD‐inhibitor DETC increases AT1 receptor mRNA expression in HK2 cells: Cells were treated with DETC (500 μm, 2 h) and H_2_O_2_ (50 μm, 30 min) in the absence and presence of tempol (1 mm, 2 h, cells were pretreated with tempol for 10 min) and AT1 receptor mRNA was determined by RT‐qPCR (details in Methods). Reference gene 18S was used to normalize the data. Bars represent results as mean ± SEM. *N* = 4 separate experiments, *P* < 0.008, *significantly different from control, ^#^significantly different from DETC.

As shown in Fig. 5, DETC treatment significantly increased the mRNA levels of AT1 receptor compared to the control cells. This effect was attenuated with tempol treatment. However, H_2_O_2_ did not show any effect on the AT1 receptor mRNA levels (Fig. 5).

### Effect of SOD‐inhibitor DETC on AT1 receptor protein expression in HK2 cells

**Figure 6:**
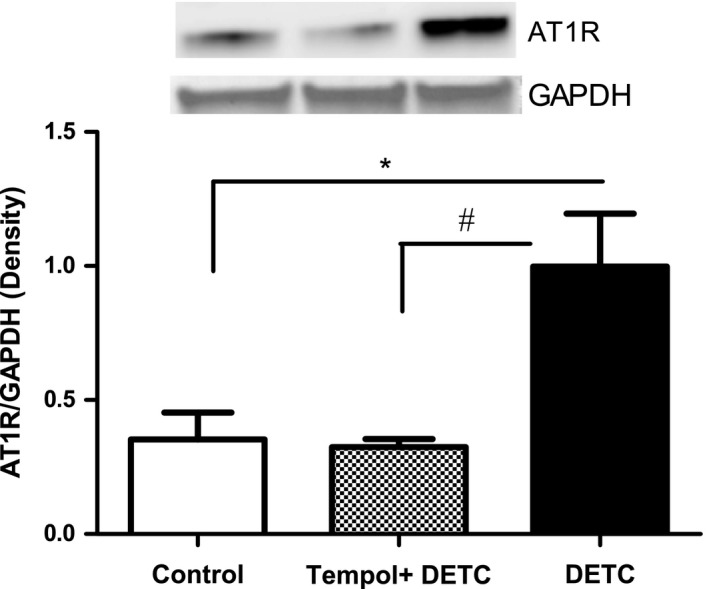
SOD‐inhibitor DETC increases AT1 receptor protein levels in HK2 cells: Cells were treated with DETC (500 μm, 2 h) in the absence and presence of tempol (1 mm, 2 h, cells were pretreated with tempol for 10 min) and cell lysate was used to measure AT1 receptor protein by immunoblotting (details in Methods). Upper panel: representative blot of AT1 receptor and loading control GAPDH. Lower panel: bars represent ratios of the densities of AT1 receptor and GAPDH proteins bands. Results are mean ± SEM. *N* = 4 separate experiments, *P* < 0.008, *significantly different from control, ^#^significantly different from DETC.

Diethyldithiocarbamate treatment significantly increased the expression levels of AT1 receptor protein compared to the control. This effect was attenuated by tempol treatment (Fig. 6).

### Effect of Sp3 overexpression on AT1 receptor protein expression in HK2 cells

**Figure 7:**
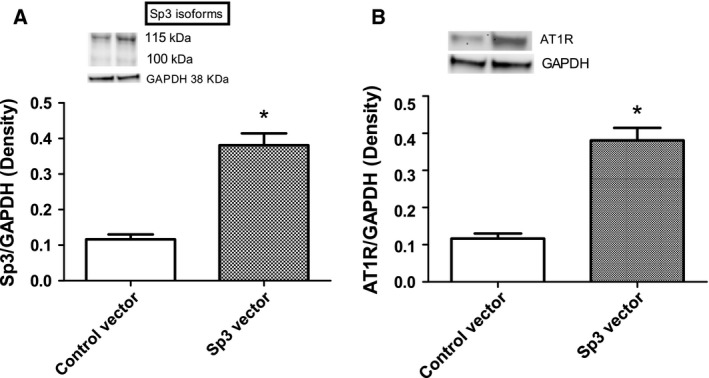
Sp3 overexpression increases AT1 receptor protein expression in HK2 cells: HK2 cells were transfected with Sp3 vector (1 μg, 24 h) and cell lysate was used to measure Sp3 (A) and AT1 receptor (B) proteins by western blot (details in Methods). Upper panels: representative blots of Sp3 (A), AT1 receptor (B), and protein loading controls GAPDH (A, B). Lower panels: bars represent ratios of the densities between Sp3 and GAPDH (A) and AT1 receptor and GAPDH (B). Both the Sp3 protein bands were considered for quantification (A). Results are mean ± SEM. *N* = 4 separate experiments (A, B), *P* < 0.003, *significantly different from control vector (A, B).

Sp3 vector‐transfected cells showed significantly increased expression of Sp3 protein compared to the control cells confirming its overexpression (Fig. 7A). Sp3 overexpression also significantly increased the levels of AT1 receptor proteins compared to control vector transfected cells (Fig. 7B).

### Effect of Sp3 siRNA on the expression of AT1 receptor protein in HK2 cells

**Figure 8:**
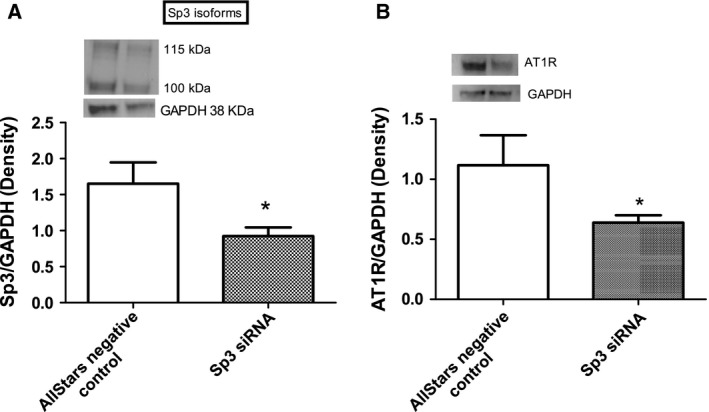
Sp3 siRNA decreases AT1 receptor protein expression in HK2 cells: HK2 cells were transfected with either Sp3 or control siRNA (100 ng, 24 h) and cell lysate was used to measure protein levels of Sp3 (A) and AT1 receptor (B) by western blotting (details in Methods). Upper panels: representative blots of Sp3 (A), AT1 receptor (B), and protein loading control GAPDH (A, B). Lower panels: bars represent ratios of the densities between Sp3 and GAPDH (A) and AT1 receptor and GAPDH (B). Both the Sp3 protein bands were considered for quantification (A). Results are mean ± SEM. *N* = 4 separate experiments (A, B), *P* < 0.01, *significantly different from AllStars negative control (A, B).

Sp3 siRNA transfection significantly reduced the expression levels of Sp3 and AT1 receptor proteins compared to control siRNA‐treated cells (Fig. 8A,B).

### Effect of Sp3 siRNA on DETC‐induced AT1 receptor protein expression in HK2 cells

**Figure 9:**
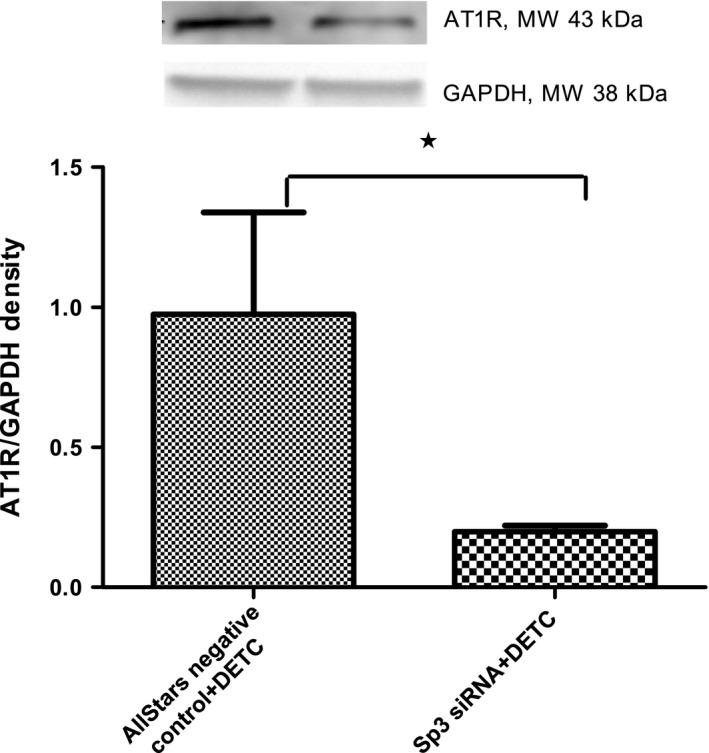
SOD‐inhibitor DETC via Sp3 regulates AT1 receptor protein expression in HK2 cells: Cells were transfected with Sp3 or control siRNA (100 ng, 24 h) followed by DETC treatment (500 μm, 2 h) and cell lysate was used to measure protein levels of AT1 receptor by western blotting. Upper panel: representative blots of AT1 receptor and loading control GAPDH. Lower panel: bars represent ratios of the densities of AT1 receptor and GAPDH protein bands. Results are mean ± SEM. *N* = 4 separate experiments, *P* < 0.008, *significantly different from control.

Diethyldithiocarbamate increased the levels of AT1 receptor protein in the control siRNA‐transfected compared to the Sp3 siRNA‐transfected cells (Fig. 9).

### Effect of SOD‐inhibitor DETC on cell membrane AT1 receptor levels in HK2 cells

**Figure 10:**
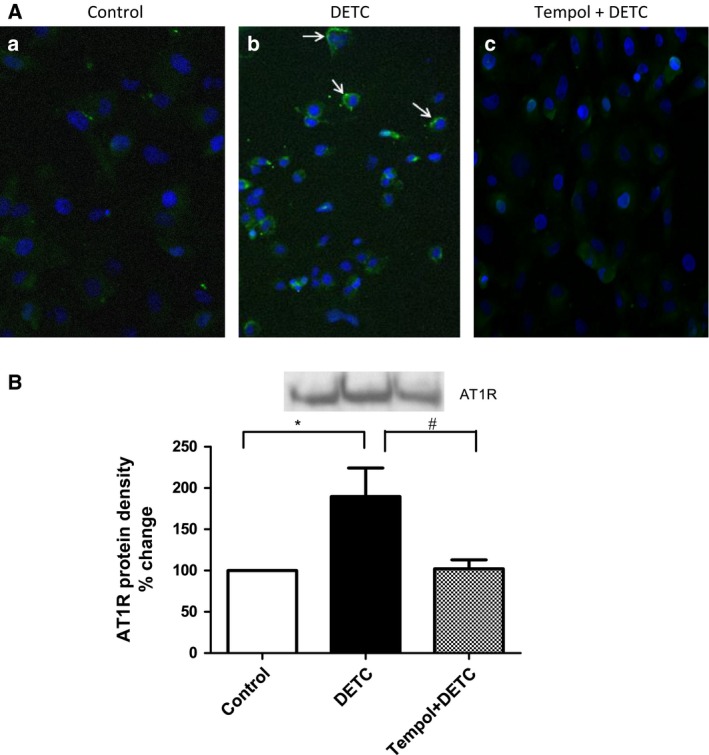
SOD‐inhibitor DETC increases cell membrane AT1 receptor in HK2 cells: Cells were treated with DETC (500 μm, 2 h) in the absence and presence of tempol (1 mm, 2 h, cells were pretreated with tempol for 10 min) (details in Methods). (A) Cells were fixed with formalin followed by labeling with primary AT1 receptor and secondary Alexa488‐conjugated antibodies. Cells were mounted with media containing nuclear dye DAPI. Arrows in panel b show cell membrane AT1 receptor (green). Nuclei are blue stained with DAPI (panels: a, b and c). *N* = 4 separate experiments. (B) Cell membrane proteins were biotinylated and isolated with avidin‐conjugated magnetic beads followed by measuring AT1 receptor proteins by western blotting. Upper panel: representative blot of AT1 receptor. Lower panel: bars represent the densities of AT1 receptor protein bands. Results are mean ± SEM. *N* = 4 separate experiments, *P* < 0.05, *significantly different from control, ^#^significantly different from DETC.

Diethyldithiocarbamate treatment caused an increase in AT1 receptor protein on the cell membrane as detected by immunofluorescence imaging (Fig. 10A) and western blotting (Fig. 10B). Tempol treatment attenuated the effects of DETC (Fig. 10A,B).

### Effect of SOD‐inhibitor DETC on PKC activity in HK2 cells

**Figure 11:**
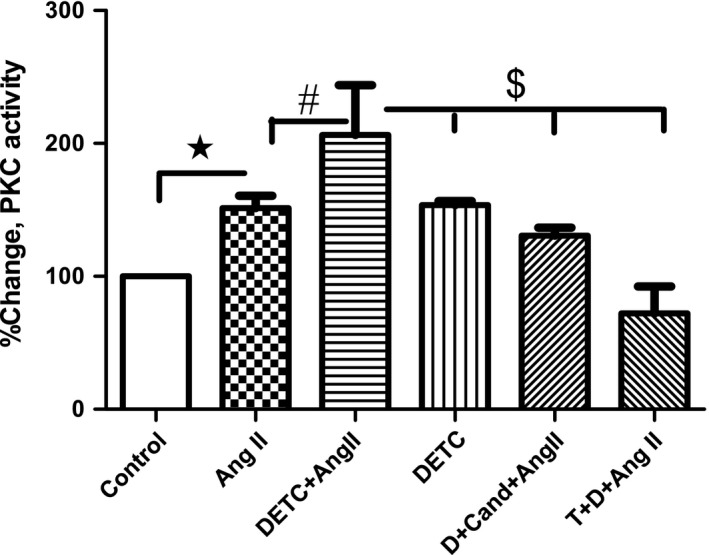
SOD‐inhibitor DETC increases angiotensin II‐mediated PKC activity in HK2 cells: Cells were treated with vehicle (control) or DETC (500 μm, 2 h) in the absence and presence of candesartan (Cand, 1 μm, 2 h) and tempol (1 mm, 2 h). Cells were pretreated with tempol and Cand for 10 min before adding DETC. Thereafter, cells were treated with or without angiotensin II (Ang II, 1 μm, 10 min), and cell lysate was used for PKC activity (details in Methods). Results are mean ± SEM. *N* = 4–6 separate experiments, *P* < 0.05, *significantly different from control, ^#^significantly different from Ang II, ^$^significantly different from DETC plus Ang II.

Compared to the vehicle, angiotensin II (1 μm) treatment increased PKC activity in control (absence of DETC) cells. Angiotensin II further increased PKC activity in DETC‐treated cells (Fig. 11). The effect of angiotensin II on PKC was attenuated by AT1 receptor blocker candesartan and SOD‐mimetic tempol (Fig. 11) in DETC‐treated cells.

## Discussion

Various studies have shown that ROS up‐regulate AT1 receptor function that presumably contributes to hypertension 4, 5, 7. However, the mechanism(s) and specificity of ROS (termed oxidative stress) associated up‐regulation of AT1 receptor function has never been addressed. To our knowledge, this study is the first to clarify the role of one type of ROS, namely superoxide, in increasing the expression and function of AT1 receptor in human kidney cells. Moreover, our studies further confirm that DHE and DCFHDA are specific fluorescent probes for superoxide and H_2_O_2_ detection, respectively.

Effects of different treatments, including DETC, tempol, and H_2_O_2_, in HK2 cells, were evaluated by toxicity measurements. The drugs used in the study did not reduce cell viability. Rather DETC increased cell viability which was reduced with tempol (superoxide effect). We do not know the reasons for this, which needs to be determined in future. These data suggest that the concentrations of DETC and H_2_O_2_ used in our studies do not reduce cell viability. Our results with superoxide (DETC‐induced) treatment demonstrated that it caused nuclear accumulation of redox‐sensitive transcription factor Sp3. In contrast, H_2_O_2_ failed to increase nuclear accumulation of Sp3. Interestingly, superoxide (DETC)‐mediated Sp3 activation correlated with an increase in AT1 receptor mRNA and protein expression in HK2 cells, while H_2_O_2_ failed to cause Sp3 activation or augmented AT1 receptor expression. While brief drug treatments are reported to induce transcription process in the cell culture systems 30, 31, the possibility of DETC affecting the stability of AT1R mRNA and protein cannot be precluded in the present study. Further studies are needed to determine if DETC affects the stability of AT1 receptor mRNA and protein.

Here, although the specific mechanism of DETC‐induced nuclear translocation of Sp3 is not completely understood, nuclear accumulation of transcription factors in general is an index of their activity. However, there are reports attributing lysine acetylation for the activation and nuclear translocation of Sp3 32, which in turn could be the one induced by superoxide. Human AT1 receptor gene possesses putative binding sites for Sp3 transcription factor 26 and Sp family transcription factors have been shown to regulate basal expression of human AT1 receptor gene 33, 34.

We validated the specificity of AT1 receptor antibody (SCBT) used in these studies prior to its application. Western blotting image indicated depleted expression of AT1 receptor protein in AT1 receptor siRNA‐treated HK2 cells. We further validated the specificity of the antibody in HEK cells which do not express AT1R proteins. We found that the antibody did not detect AT1R protein in HEK compared to HK2 cells. Importantly, AT1 receptor antibody did not give a nonspecific, false‐positive signal either in the siRNA‐treated cells or in the HEK cells, which ratifies its specificity and hence its further use. Studies by Patel *et al*. also confirmed the absence of any nonspecific binding by this antibody 29.

Based on our results with DETC, it seems reasonable to suggest that superoxide (DETC‐induced) increases the expression levels of renal AT1 receptor via Sp3 transcription factor activation. Relevant to this, our transfection studies in HK2 cells showed that Sp3 overexpression increased AT1 receptor protein expression, while Sp3 siRNA decreased the expression levels of both Sp3 and AT1 receptor proteins. Furthermore, a decrease in AT1 receptor protein lower than that of control cells with Sp3 siRNA corroborates previous studies 33, 34, indicating a role for Sp3 in basal expression of AT1 receptor even in the absence of external stress factors (ROS). Moreover, superoxide (DETC‐induced) increased mRNA and protein levels of AT1 receptor and the increased expression and function of AT1 receptor caused by superoxide were attenuated by tempol treatment. Increasing evidence from our own laboratory and others suggests that oxidative stress increases membrane receptor number and thereby the function of AT1 receptor 35, 36, which consequently contributes to hypertension 4, 5 and potentially other cardiovascular diseases 9. In connection with this, our immunostaining and biotinylation studies show that superoxide (DETC‐induced) was responsible for increasing membranous AT1 receptor levels (25% increase with biotinylation) in HK2 cells. This phenomenon might be potentially responsible for higher AT1 receptor function in hypertension. Relevant to this, we found higher AT1 receptor function, measured as AT1 receptor ligand‐mediated (Ang II) increase in PKC activity in DETC‐treated (superoxide) cells compared to the control. We also found that DETC alone (superoxide effect) increased PKC activity, which is in agreement with the previous reports that ROS activate PKC activity, 37, 38.

ROS, specifically superoxide and H_2_O_2_, are increasingly recognized as key signaling molecules 9, 39. Many studies in different rat models (aging rats, spontaneously hypertensive rats, salt‐sensitive Dahl rats, and cyclosporine‐induced rat model) have implicated renal ROS in the pathogenesis of hypertension 4, 5, 17, 40. Clinical studies have also shown that high levels of ROS cause hypertension in humans 41, 42, 43, 44, although some clinical studies are inconsistent 44, 45. However, a specific role of individual ROS has never been addressed. The present study suggests that superoxide is perhaps the ROS that is involved in increased AT1 receptor function in renal cells.

## Conclusions

This study demonstrates that superoxide, but not H_2_O_2_, up‐regulates renal AT1 receptor function via transcription factor Sp3 and thus provides a mechanism of regulation for renal AT1 receptor. Specific targeting of superoxide and/or Sp3 may provide a selective and better therapeutic target to combat oxidative stress and/or its mediated effects responsible for hypertension and associated cardiovascular diseases. HK2 cells may also offer a good *in vitro* model system to understand the mechanistic aspects of kidney physiology. However, they are not a replica of renal proximal cells and have limitations such as lack of important apical and basolateral transporters 46. Hence, while current studies are valuable in understanding the general regulation of renal AT1 receptor expression, further research needs to be conducted to extend their clinical significance.

## Author contributions

MS: acquisition, analysis, and interpretation of data, preparation of manuscript. IP: drafting and critical revision of the article. MA: conception and design of the study and final approval of manuscript.

## Supporting information


**Fig. S1.** Nuclear fraction is devoid of cytoplasmic contamination.
