# Supplementary material for: Superoxide increases angiotensin II AT1 receptor function in human kidney‐2 cells
Source: FEBS Open Bio. 2016 Nov 16;6(12):1273–84. doi: 10.1002/2211-5463.12148 (PMC5302058; doi:10.1002/2211-5463.12148)
Supplement: Supplementary file 1 — Fig. S1. Nuclear fraction is devoid of cytoplasmic contamination. [file FEB4-6-1273-s001.pdf]

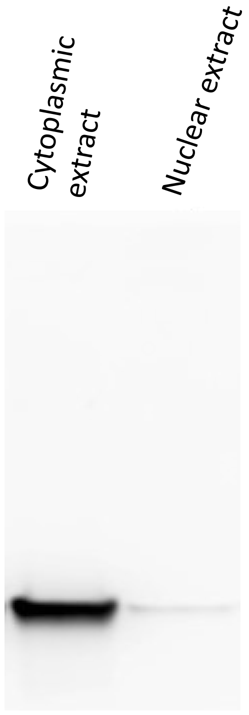

Supplemental Figure 1: Nuclear fraction is devoid of cytoplasmic contamination: Adherent HK2 cells were harvested with trypsin EDTA, washed in PBS and pelleted. Nuclear and cytoplasmic extracts were separated by a kit based method (Thermo Fisher Scientific, Catalog # 78835) following manufacturer's instructions as described in Methods. Purity of the fractions was assessed by western blotting of GAPDH. Representative blot of GAPDH in cytoplasmic and nuclear fractions.
